# Cobb angle changes after standardized chiropractic intervention in 12 females with adolescent idiopathic scoliosis with double major curve types: a retrospective review of patient records

**DOI:** 10.1186/1748-7161-8-S2-P5

**Published:** 2013-09-18

**Authors:** Alan Woggon, Daniel Martinez

**Affiliations:** 1 CLEAR Scoliosis Institute, St Cloud, MN, USA

## Background

Scoliosis is a 3-dimensional spinal deformity measured in two dimensions. [1] and is the leading orthopedic problem seen in school-aged children, affecting approximately 2-4% of children 10 to 16 years of age. First introduced in 1948, the Cobb angle is the current standard for measuring the severity of scoliosis. As stated by Gstoettner et al. in 2007, “It is an objective measure and is generally used to make decisions about the progression of a curve, as well as the need and success of treatment. Measurements of the Cobb angle bare an intra- and interobserver variability of approximately 4° to 8°. The definition of end vertebrae introduces the main source of error.”[2]

## Methods

Procedures - The authors reviewed the clinical records of 140 consecutive patients who presented for treatment of their scoliosis at a private chiropractic practice from June 2007 to February 2008, and selected to report the Cobb angle changes in adolescent females with idiopathic scoliosis and double major (right thoracic, left lumbar) curve patterns; 12 patients fit this criteria. All patients were negative for malignancy, fracture, previous arthrodesis and scoliosis secondary to congenital or pathologic disorders. Informed consent to treatment, radiography, and to the collection of data for research purposes was obtained from all patients. Treatment - The treatment provided to the patients in this study included a standardized intervention over a 2-week period, consisting of soft tissue rehabilitation, chiropractic manipulative therapy, and neuromuscular re-education. The average length of one treatment session was between 150 and 180 minutes. 

## Results

Cobb angles were drawn using the same end vertebrae on pre- and post-treatment scoliosis radiographs. Changes of 8° or less were not considered significant. Out of 12 cases, a change of greater than 8° occurred in nine thoracic Cobb angles and ten lumbar Cobb angles. 

## Conclusions and discussion

The changes in Cobb angles reported in this study could be due to either a short-term benefit of the applied intervention or to the wide variance in Cobb angle that may occur within a short timeframe. Due to this uncertainty, the significance of these results should be considered meaningful only to the individuals involved in the study. There are several weaknesses to using the Cobb angle as the primary measure to determine the need for or success of scoliosis treatment, such as its high measurement error, 2-dimensional nature, possible wide variance within a short timeframe and poor correlation with functional and cosmetic indices. Gstoettner et al. state, “One pitfall in scoliosis measurement implies the Cobb method itself. Until we develop a proper tri-dimensional measuring system, no matter how good the antero-posterior and lateral imaging results are, it is still only a two-dimensional picture.”[2] Beauchamp et al. reported a high variance in the measurement of Cobb angle within a single day. [3] Despite these shortcomings, it remains the standard by which the success (or failure) of scoliosis treatment is determined.
